# Obesity and lipid-related parameters for predicting metabolic syndrome in Chinese elderly population

**DOI:** 10.1186/s12944-018-0927-x

**Published:** 2018-12-20

**Authors:** Zhan Gu, Ping Zhu, Qiao Wang, Huayu He, Jingjuan Xu, Li Zhang, Dong Li, Jianying Wang, Xiaojuan Hu, Guang Ji, Lei Zhang, Baocheng Liu

**Affiliations:** 1 Shanghai Innovation Center of TCM Health Service, Shanghai University of Traditional Chinese Medicine, Shanghai, 201203 China; 2 School of Rehabilitation Science, Shanghai University of Traditional Chinese Medicine, Shanghai, 201203 China; 3 Zhangjiang Community Health Service Center of Pudong New District, Shanghai, 201210 China; 4 Institute of Digestive Diseases, China-Canada Center of Research for Digestive Diseases (ccCRDD), Longhua Hospital, Shanghai University of Traditional Chinese Medicine, Shanghai, 200032 China

**Keywords:** Metabolic syndrome, Obesity and lipid-related parameters, Chinese elderly population, Lipid accumulation product, Visceral adiposity index, Triglyceride-to-high-density-lipoprotein-cholesterol, Waist-to-height ratio, Body mass index

## Abstract

**Background:**

The present study evaluated the predictive ability of five known “best” obesity and lipid-related parameters, including body mass index (BMI), waist-to-height ratio (WHtR), triglyceride-to-high-density-lipoprotein-cholesterol (TG/HDL-C), lipid accumulation product (LAP) and visceral adiposity index (VAI), in identifying metabolic syndrome (MetS) in Chinese elderly population.

**Methods:**

A total of 6722 elderly Chinese subjects (≥60 years) were recruited into our community-based cross-sectional study from April 2015 to July 2017. The anthropometrics, blood pressure, fasting plasma glucose, blood lipid profiles, family history and health-related behaviours were assessed.

**Results:**

The prevalence of MetS was 40.4% (32.5% in males and 47.2% in females). With the increase in the number of MetS components (from 0 to 5), all the five parameters showed an increase trend in both genders (all *P* for trend < 0.001). According to receiver operating characteristic curve (ROC) analyses, all the five parameters performed high predictive value in identifying MetS. The statistical significance of the areas under the curves (AUCs) differences suggested that the AUCs of LAP were the greatest among others in both genders (AUCs were 0.897 in males and 0.875 in females). The optimal cut-off values of LAP were 26.35 in males and 31.04 in females. After adjustment for potentially confounding factors, LAP was strongly associated with the odds of having MetS in both genders, and ORs for MetS increased across quartiles using multivariate logistic regression analysis (*P* < 0.001).

**Conclusion:**

LAP appeared to be a superior parameter for predicting MetS in both Chinese elderly males and females, better than VAI, TG/HDL-C, WHtR and BMI.

## Background

Metabolic syndrome (MetS) is a complex of interrelated risk factors for cardiovascular disease (CVD) and diabetes. These factors include central obesity, elevated blood pressure (BP), elevated fasting plasma glucose (FPG) levels, elevated triglyceride (TG) levels, and reduced high-density lipoprotein cholesterol (HDL-C) levels [1–3]. As a result of rapid changes in lifestyle and dietary habits, MetS has recently become an epidemic threatening the health of Chinese people, especially those elderly ones. A systematic review has shown that the prevalence of MetS is 24.5% in China, and it is higher (32.4%) in Chinese elderly [4]. Additionally, a national study in 2014–2015 found that the prevalence of MetS was higher in the Chinese elderly and females [5]. Hence, early identification of subjects at high risk of MetS is imperative to prevent occurence and progression of MetS.

Obesity and blood lipids are closely related to CVD and diabetes. Various obesity and lipid-related parameters, especially body mass index (BMI), waist-to-height ratio (WHtR), triglyceride-to-high-density-lipoprotein-cholesterol (TG/HDL-C), lipid accumulation product (LAP) and visceral adiposity index (VAI), have commonly been used to predict MetS and each one of these five parameters has ever been reported as the “best” parameter for predicting MetS in previous studies [6–12]. Nevertheless, there has been no study on all these five known “best” parameters for predicting MetS to date via PubMed database (https://www.ncbi.nlm.nih.gov/pubmed) and it has still been argued about which parameter conveys the highest risk of MetS.

BMI is a measurement of body fat based on height and weight [13]. WHtR reflects abdominal or central obesity. BMI and WHtR have been regarded as two powerful anthropometric parameters for detection of cardiometabolic risk factors in previous studies [6, 7, 14, 15]. Evidence has shown that lipid ratios perform better than individual lipids in predicting cardiometabolic risk and TG/HDL-C is considered to be a better marker to identify MetS [8, 9]. LAP and VAI are two recent indicators and are used to estimate visceral obesity. LAP is calculated as a combination of waist circumference (WC) and TG [16]. VAI, introduced by the AlkaMeSy Study Group, comprises anthropometric parameters (BMI and WC) and lipid parameters (TG and HDL-C) [17]. LAP and VAI have been proposed as simple and useful markers of MetS in recent studies [10–12].

It is important to identify a parameter to serve as a quick and powerful tool for prevention and simple diagnosis of MetS in Chinese elderly population. Therefore, the purpose of our study is to compare the predictive ability of these five “best” obesity and lipid-related parameters for identifying males and females with MetS in elderly Chinese subjects.

## Methods

### Study subjects

This study is a community-based cross-sectional investigation for the elderly population in Shanghai, China. A total of 7098 residents from Zhangjiang community of Shanghai aged ≥60 years were recruited into our study from April 2015 to July 2017. The study was performed according to the guidelines of the Helsinki Declaration. A standard protocol was designed by Shanghai innovation center of TCM health service and was approved by the Ethics Committee of Shanghai University of Traditional Chinese Medicine. Written informed consent was obtained from all subjects.

The inclusion criteria included age ≥ 60 years, local residents in Shanghai, complete data measurements and informed consents. Subjects with mental disorders, malignant tumors or incomplete recorded information were excluded from this project based on their medical records. After investigation, 376 subjects were excluded from the study. A total of 6722 Chinese elderly subjects (3077 males and 3645 females) with complete data were finally included in this study.

### Data measurements

The obesity and lipid-related parameters included BMI, WHtR, TG/HDL-C, LAP and VAI. Height and weight were measured to the nearest 0.1 cm and 0.1 kg using electronic measurement instrument (Shengyuan; Zhengzhou, China). WC was measured to the nearest 0.1 cm using a flexible metric measuring tape (Pudong CDC; Shanghai, China). All subjects were measured wearing light clothing without hats and shoes. Blood pressure was measured with electronic sphygmomanometers (Biospace; Cheonan, South Korea) using the standard recommended procedures. Blood samples were obtained from the antecubital vein in the morning after an overnight fasting period. FPG, TG, HDL-C, low-density lipoprotein cholesterol (LDL-C) and total cholesterol (TC) were measured using an automatic biochemistry analyzer (Hitachi; Tokyo, Japan). Current smoking (current smokers or non-smokers), alcohol consumption (currently consume alcohol or do not consume alcohol) and family history of CVD (yes or no) were recorded by self-report.

BMI was calculated as bodyweight (kg)/height^2^ (m^2^). WHtR was calculated as WC (cm)/height (cm). TG/HDL-C was calculated as TG (mmol/L)/HDL-C (mmol/L). LAP and VAI were calculated based on the gender-specific mathematical model formula. LAP = [WC (cm) - 65] × TG (mmol/L) for males, and [WC (cm) - 58] × TG (mmol/L) for females [16]. WC values ≤65 cm in males and ≤ 58 cm in females were revised upward to 66.0 cm and 59.0 cm to avoid invalid data [16]. VAI = [WC (cm)/[39.68 + (1.88 × BMI)]] × [TG (mmol/L)/1.03] × [1.31/HDL-C (mmol/L)] for males, and [WC (cm)/[36.58 + (1.89 × BMI)]] × [TG (mmol/L)/0.81] × [1.52/HDL-C (mmol/L)] for females [17].

### Definition of MetS

According to the diagnosis criteria proposed by the International Diabetes Federation (IDF) and the American Heart Association (AHA)/National Heart, Lung and Blood Institute (NHLBI) in 2009 [1] combined with an amended definition of abdominal obesity for Chinese population [18], MetS was defined as the presence of three or more of those following features: (a) central obesity: WC ≥ 90 cm for males or ≥ 85 cm for females; (b) elevated BP: systolic blood pressure (SBP) ≥ 130 mmHg or diastolic blood pressure (DBP) ≥ 85 mmHg, or ongoing antihypertensive medications; (c) elevated FPG: FPG ≥ 5.6 mmol/L, or ongoing anti-diabetic treatment; (d) elevated TG: TG ≥ 1.7 mmol/L; and (e) reduced HDL-C: HDL-C < 1.0 mmol/L in males and < 1.3 mmol/L in females.

### Statistical analyses

All of the descriptive statistics for all of the variables were calculated. Categorical variables were expressed as counts or percentages and compared using Pearson’s χ^2^ tests. Continuous variables were expressed as mean ± standard deviation and compared using two-sided *t* tests. One-way ANOVA test was used for comparisons of the levels of obesity and lipid-related parameters across number of MetS components. To compare the predictive ability and determine the optimal cut-off values of the parameters for predicting MetS, receiver operating characteristic curve (ROC) analyses were used. The areas under the receiver operating characteristic curves (AUCs) were calculated, and the optimal cut-off values were identified from the maximum Youden index (sensitivity plus specificity - 1) to determine the appropriate parameters. Multivariate logistic regression analysis was applied to calculate odds ratios (ORs) and 95% confidence intervals (CIs) for MetS across quartiles of the parameters, with quartile 1 as reference group, adjusting for potentially confounding factors such as age, current smoking, alcohol consumption and family history of CVD. Considering significant gender differences in body fat distribution and MetS [19, 20], all statistical analyses were separately performed within males and females.

*P* values < 0.05 were set as significant for all of the statistical tests for bilateral contrasts. All statistical analyses were conducted using SPSS version 21.0 (SPSS; Chicago, USA). The statistical significance of the differences in the AUCs was analyzed using MedCalc version 17.1.0 (MedCalc; Ostend, Belgium) with the algorithm developed by DeLong’s research team [21].

## Results

### Baseline characteristics

As shown in Table 1, the subjects with MetS tended to have significantly higher BMI, WC, WHtR, SBP, DBP, FPG, TG, TG/HDL-C, LAP and VAI, and lower HDL-C compared with the subjects without MetS in both genders (*P* < 0.001). The subjects with MetS had higher prevalence of current smoking and alcohol consumption compared with the subjects without MetS in both genders (*P* < 0.05). Age, LDL-C and the prevalence of family history of CVD were not significantly different between males with MetS and without MetS. TC was higher in males with MetS compared to without MetS (*P* < 0.05). Age and the prevalence of family history of CVD were higher in females with MetS compared to without MetS (*P* < 0.001). TC and LDL-C were higher in females without MetS compared to with MetS (*P* < 0.05).

**Table 1:** The baseline characteristics of the study subjects

Variable	Male (*n* = 3077)			Female (*n* = 3645)		
	With MetS (*n* = 999)	Without MetS (*n* = 2078)	*P* value	With MetS (*n* = 1719)	Without MetS (*n* = 1926)	*P* value
Age (years)	70.02 ± 7.21	69.98 ± 7.42	0.885	70.59 ± 7.51	69.70 ± 7.75	< 0.001
Current smoking	94 (9.41%)	129 (6.21%)	0.001	17 (0.99%)	5 (0.26%)	0.005
Alcohol consumption	260 (26.03%)	242 (11.65%)	< 0.001	172 (10.01%)	75 (3.89%)	< 0.001
Family history of CVD	249 (24.92%)	513 (24.69%)	0.886	535 (31.12%)	423 (21.96%)	< 0.001
BMI (kg/m^2^)	26.02 ± 2.83	23.14 ± 3.05	< 0.001	25.74 ± 3.77	22.58 ± 3.04	< 0.001
WC (cm)	90.6 ± 7.8	81.2 ± 7.8	< 0.001	85.8 ± 8.6	76.7 ± 7.9	< 0.001
WHtR	0.54 ± 0.05	0.49 ± 0.05	< 0.001	0.55 ± 0.06	0.50 ± 0.05	< 0.001
SBP (mmHg)	149 (136, 162)	136 (122, 153)	< 0.001	148 (136, 161)	134 (120, 151)	< 0.001
DBP (mmHg)	84.6 ± 11.5	79.7 ± 12.3	< 0.001	84.9 ± 11.2	79.8 ± 12.1	< 0.001
FPG (mmol/L)	6.3 (5.7, 7.4)	5.5 (5.1, 6.0)	< 0.001	6.0 (5.6, 6.9)	5.4 (5.1, 5.8)	< 0.001
TC (mmol/L)	4.81 (4.21, 5.45)	4.72 (4.16, 5.33)	0.016	5.27 (4.64, 5.91)	5.36 (4.75, 5.94)	0.003
TG (mmol/L)	2.11 ± 1.57	1.09 ± 0.56	< 0.001	2.03 ± 1.35	1.15 ± 0.49	< 0.001
HDL-C (mmol/L)	1.02 ± 0.20	1.25 ± 0.25	< 0.001	1.17 ± 0.20	1.46 ± 0.27	< 0.001
LDL-C (mmol/L)	3.01 (2.47, 3.56)	3.00 (2.47, 3.54)	0.795	3.24 (2.66, 3.85)	3.37 (2.77, 3.89)	0.005
TG/HDL-C	2.20 ± 2.07	0.93 ± 0.60	< 0.001	1.88 ± 1.65	0.84 ± 0.48	< 0.001
LAP	51.71 ± 38.60	18.27 ± 13.53	< 0.001	55.25 ± 40.15	21.75 ± 13.16	< 0.001
VAI	2.84 ± 2.61	1.16 ± 0.75	< 0.001	3.54 ± 3.16	1.52 ± 0.88	< 0.001

Data are expressed as mean ± standard deviation, median (interquartile range 25–75%), or counts (percentages)

*MetS* metabolic syndrome, *CVD* cardiovascular disease, *BMI* body mass index, *WC* waist circumference, *WHtR* waist-to-height ratio, *SBP* systolic blood pressure, *DBP* diastolic blood pressure, *FPG* fasting plasma glucose, *TC* total cholesterol, *TG* triglyceride, *HDL-C* high-density lipoprotein cholesterol, *LDL-C* low-density lipoprotein cholesterol, *LAP* lipid accumulation product, *VAI* visceral adiposity index

### Prevalence of MetS

**Fig. 1:**
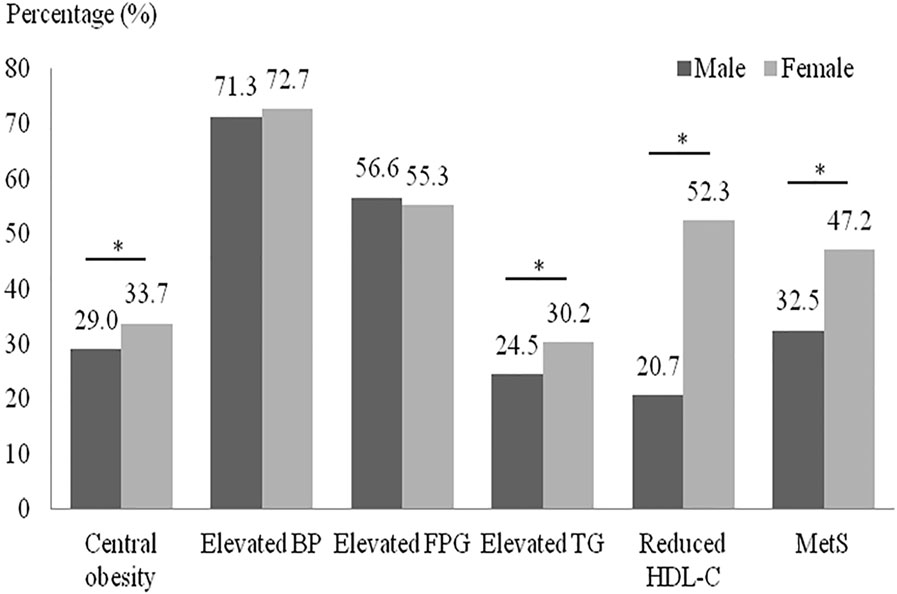
Prevalence of MetS and its individual components in males and females (* *P* < 0.001)

Prevalence of MetS and its individual components in Chinese elderly males and females are shown in Fig. 1. Approximately 40.4% of the subjects had MetS (32.5% in males and 47.2% in females). The prevalence of MetS in females was significantly higher than that in males (*P* < 0.001). Among the individual components of MetS, the prevalence of central obesity, elevated TG and reduced HDL-C in females were significantly higher than that in males (*P* < 0.001). The prevalence of elevated BP and elevated FPG were not significantly different between males and females.

### Levels of the parameters across number of MetS components

Levels of the five obesity and lipid-related parameters in the study subjects across number of MetS components are listed in Table 2. With the increase in the number of MetS components (from 0 to 5), BMI, WHtR, TG/HDL-C, LAP and VAI all showed an increase trend in both genders (all *P* for trend < 0.001).

**Table 2:** Levels of the five parameters across number of MetS components

Variable	0	1	2	3	4	5	*P* for trend
Male	(n = 307)	(*n* = 796)	(*n* = 975)	(*n* = 611)	(*n* = 298)	(*n* = 90)	
BMI	21.77 ± 2.60	22.66 ± 3.11	23.97 ± 2.89	25.68 ± 2.84	26.26 ± 2.69	27.55 ± 2.61	< 0.001
WHtR	0.47 ± 0.04	0.48 ± 0.04	0.50 ± 0.05	0.54 ± 0.05	0.55 ± 0.04	0.57 ± 0.04	< 0.001
TG/HDL-C	0.74 ± 0.30	0.85 ± 0.43	1.05 ± 0.74	1.70 ± 1.19	2.65 ± 1.97	4.07 ± 3.40	< 0.001
LAP	11.86 ± 8.32	15.70 ± 10.87	22.39 ± 15.39	39.76 ± 20.32	62.16 ± 34.16	98.27 ± 80.30	< 0.001
VAI	0.91 ± 0.38	1.05 ± 0.54	1.32 ± 0.93	2.17 ± 1.41	3.44 ± 2.47	5.36 ± 4.62	< 0.001
Female	(*n* = 256)	(*n* = 676)	(*n* = 994)	(*n* = 862)	(*n* = 631)	(*n* = 226)	
BMI	21.15 ± 2.62	21.95 ± 2.87	23.37 ± 3.02	25.07 ± 3.93	25.98 ± 3.50	27.61 ± 3.15	< 0.001
WHtR	0.47 ± 0.05	0.49 ± 0.05	0.51 ± 0.06	0.54 ± 0.06	0.56 ± 0.06	0.59 ± 0.04	< 0.001
TG/HDL-C	0.61 ± 0.24	0.74 ± 0.32	0.96 ± 0.57	1.44 ± 1.23	2.11 ± 1.60	2.91 ± 2.40	< 0.001
LAP	14.14 ± 8.25	18.38 ± 10.07	26.00 ± 14.44	39.68 ± 20.38	61.59 ± 38.48	96.96 ± 61.52	< 0.001
VAI	1.09 ± 0.43	1.33 ± 0.60	1.76 ± 1.03	2.65 ± 2.17	3.97 ± 3.00	5.71 ± 4.97	< 0.001

### The parameters for predicting MetS

**Fig. 2:**
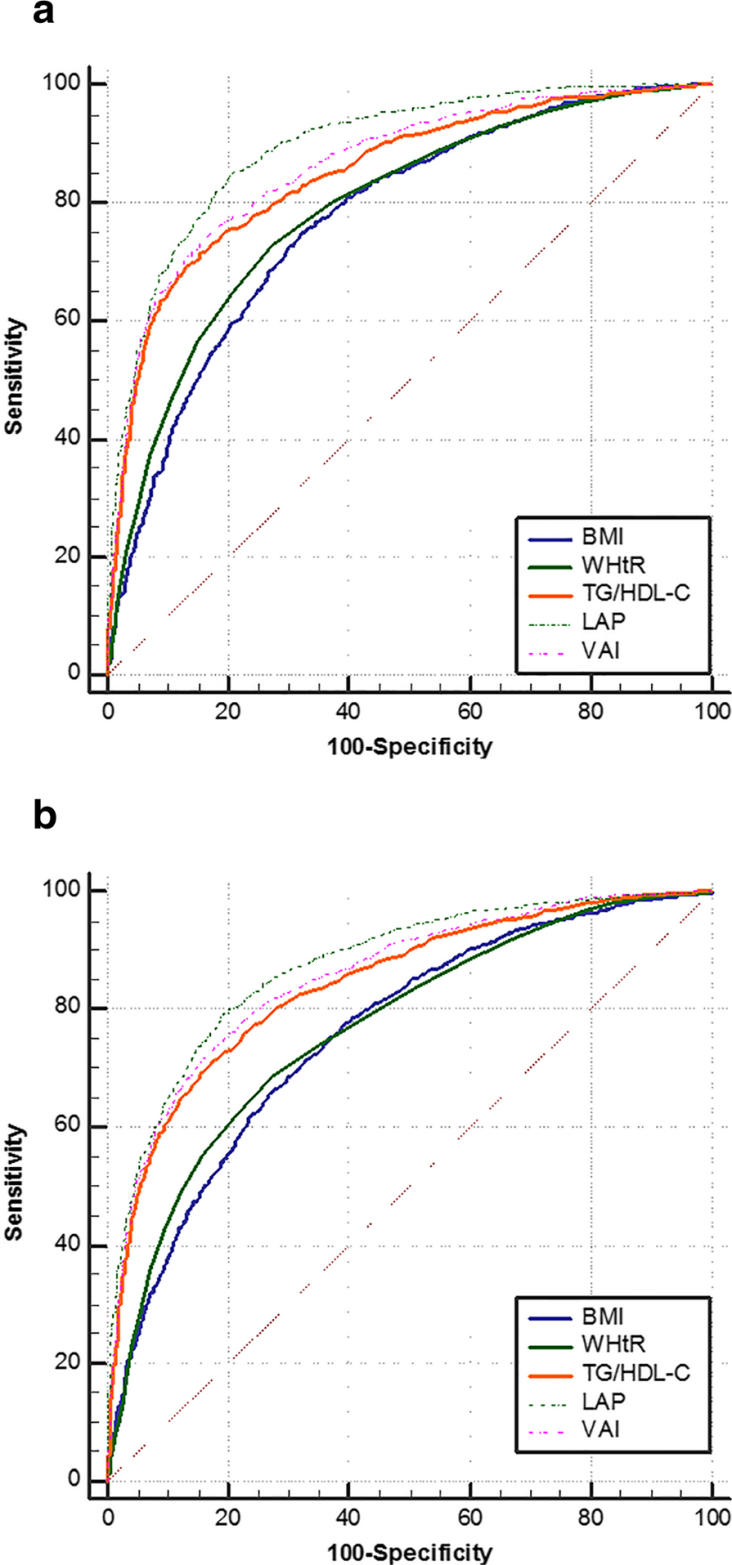
ROC curves for the five parameters to predict MetS in males (**a**) and females (**b**)

ROC curves for the five parameters to predict MetS in males and females are shown in Fig. 2. The AUCs, optimal cut-off values, sensitivity, specificity and Youden index of the five parameters for predicting MetS are shown in Table 3. The statistical significance of the AUCs differences of the five parameters in males suggested that the AUC of LAP (AUC: 0.897, 95% CI: 0.885–0.907) was the greatest among others, followed by VAI, TG/HDL-C, WHtR and BMI. In females, the AUC of LAP (AUC: 0.875, 95%CI: 0.864–0.886) was also the greatest, followed by VAI, TG/HDL-C, WHtR and BMI (WHtR and BMI did not differ). LAP had the strongest predictive ability for both genders. The optimal cut-off values of LAP were 26.35 in males and 31.04 in females.

**Table 3:** The AUCs, optimal cut-off values, sensitivity, specificity and Youden index of the five parameters for predicting MetS

Variable	AUC (95% CI)	Cut-off value	Sensitivity (%)	Specificity (%)	Youden index (%)
Male					
BMI	0.775 (0.760–0.789)	24.17	74.97	67.81	42.78
WHtR	0.791 (0.776–0.805)	0.51	72.87	72.52	45.39
TG/HDL-C	0.851 (0.838–0.864)	1.38	69.47	86.72	56.19
LAP	0.897 (0.885–0.907)	26.35	85.09	79.31	64.39
VAI	0.865 (0.853–0.877)	1.63	74.17	83.64	57.81
Female					
BMI	0.760 (0.746–0.774)	24.03	66.67	72.17	38.84
WHtR	0.769 (0.755–0.782)	0.52	68.64	72.53	41.18
TG/HDL-C	0.843 (0.831–0.855)	1.13	71.09	82.76	53.85
LAP	0.875 (0.864–0.886)	31.04	79.17	80.69	59.86
VAI	0.856 (0.844–0.867)	2.05	73.82	81.98	55.81

### MetS risk across quartiles of LAP

The adjusted ORs and 95%CI of MetS across quartiles of LAP are shown in Table 4. After adjustment for age, current smoking, alcohol consumption and family history of CVD, LAP was strongly associated with the odds of having MetS in both genders, and ORs for MetS increased across quartiles (*P* < 0.001). The study subjects in the highest quartiles of LAP had a 216.630-fold increased risk in males and a 114.291-fold increased risk in females of suffering from MetS compared with those in the reference group.

**Table 4:** The MetS risk across quartiles of LAP

	OR (95% CI)	*P* value
Male		
Q1	Reference	
Q2	4.908 (2.824–8.531)	< 0.001
Q3	29.791 (17.686–50.179)	< 0.001
Q4	216.630 (127.064–369.328)	< 0.001
Female		
Q1	Reference	
Q2	4.185 (3.169–5.528)	< 0.001
Q3	17.344 (13.203–22.785)	< 0.001
Q4	114.291 (82.115–159.075)	< 0.001

Multivariate logistic regression analysis, adjustment for age, current smoking, alcohol consumption and family history of CVD

## Discussion

In this study, we have investigated the predictive value of BMI, WHtR, TG/HDL-C, LAP and VAI, five known “best” obesity and lipid-related parameters, in identifying MetS in the Chinese elderly. The results showed that LAP performed better than the other four parameters for predicting MetS in both elderly males and females. Additionally we also confirmed LAP was strongly associated with MetS in both genders. To the best of our knowledge, this is the first comprehensive study to evaluate the predictive ability of these five “best” obesity and lipid-related parameters with MetS and to focus on a large sample of Chinese elderly population.

The prevalence of MetS is high and increasing rapidly in Chinese elderly population. A cross-sectional survey in a representative sample of elderly people in Beijing showed 58.1% of the participants had MetS and MetS was more common in females than males [22]. We found the prevalence of MetS was 40.4%, with an obviously higher prevalence was shown in females as well. As a novel marker of excessive lipid accumulation, the LAP introduced by Kahn [16] has been widely applied for recognizing cardiovascular risk over the past decade. Our study found LAP had the strongest predictive ability for identifying MetS in both genders, in line with many previous studies [10, 12, 23–27]. A recent study of 992 Chinese middle-aged and elderly subjects showed that LAP performed better than VAI and the product of triglycerides and glucose (TyG) in identifying MetS [10]. And the optimal cut-off value of LAP was 31.465 for the elderly, greater than our findings. Guo et al. suggested LAP was a better indicator for predicting MetS compared to VAI, body adiposity index (BAI) and WHtR in low income rural adults of Xinjiang, China [12]. The results showed the cut-off values of LAP were 34.7 (AUC: 0.853) in males and 27.3 (AUC: 0.817) in females. One study from Brazil also suggested that LAP had better predictive ability for MetS in the elderly and the cut-off value was 32.3 (AUC: 0.849) [28]. Our results validated the predictive value of LAP in the Chinese elderly. In addition, the strong ability of LAP for predicting MetS were also identified in Spanish, Argentinian and Iranian populations [23, 25, 27]. We also confirmed the increase of MetS risk across quartiles of LAP, consistent with both Guo et al.’s research and Ma et al.’s research [12, 29]. Moreover, LAP was also found to be a strong predictor of metabolic and related disorders, such as diabetes, insulin resistance and non-alcoholic fatty liver disease [30–32]. Indeed, LAP is a simple and quick parameter, and it only requires the WC measurement and serum TG testing. Therefore, LAP is expected to be a powerful tool and the “best” parameter among multiple obesity and lipid-related parameters to identify Chinese elderly subjects at high risk of MetS in clinical practice.

In our present study, VAI and TG/HDL-C were defined as “excellent” parameters (0.8 ≤ AUC < 0.9) based on Hosmer and Lemeshow’s criteria, while WHtR and BMI were “acceptable” parameters (0.7 ≤ AUC < 0.8). The VAI, as a surrogate marker of adipose tissue function and distribution, was independently correlated with cardiometabolic risk [17, 33, 34]. Li et al.’s research and Guo et al.’s research both suggested VAI could be applied for identifying MetS in Chinese population [10, 12], supporting our findings. One study from Iran also confirmed the predictive value of VAI was high in identifying MetS [35]. The TG/HDL-C has been proposed as a potential tool to identify patients at increased risk of MetS [8, 9, 36, 37]. A study from Canada indicated that TG/HDL-C was a superior marker to identify MetS compared to TC/HDL-C, LDL-C/HDL-C, and nonHDL-C/HDL-C based on a multiethnic sample [8]. Chen et al. proposed TG/HDL-C was a better predictor of MetS in Chinese Uighur females [9]. Consistent with these results, we also found VAI and TG/HDL-C had good predictive accuracy to identify MetS in the Chinese elderly. WHtR and BMI are two common anthropometric parameters and have been applied for detection of cardiometabolic risk for a long time. In our previous study, WHtR and BMI could be used for prediction of multiple metabolic risk factors in the Chinese elderly [14]. Yang et al. indicated WHtR was better than BMI and WC as a screening tool for MetS [7]. And Wang et al.’s research suggested BMI was useful for predicting MetS [6]. Our results found WHtR and BMI were both acceptable parameters for predicting MetS, but inferior to LAP, VAI and TG/HDL-C. Of note, our study focuses on the Chinese elderly aged 60 years and above in Shanghai, and the differences in the results with other findings may be due to the age and regional distributional differences of the samples.

There are, however, several limitations of this study. The subjects in this study were all from Shanghai, and the sample may not fully reflect all Chinese elderly. This study included quite a large number of Chinese elderly, but we only enrolled subjects who had completed the comprehensive health check study, which may have biased our primary findings. Moreover, our analyses were adjusted for age, current smoking, alcohol consumption and family history of CVD, but not for physical activity and diet known to influence obesity and lipids. Further studies with multi centers, a larger sample size and more detailed information collection are needed to assess the predictive value of obesity and lipid-related parameters in identifying elderly subjects at high risk of MetS.

## Conclusion

In conclusion, our study performed the predictive ability of obesity and lipid-related parameters, including BMI, WHtR, TG/HDL-C, LAP and VAI, in identifying MetS in Chinese elderly population. LAP appeared to be a superior parameter for predicting MetS in both Chinese elderly males and females, and was strongly associated with MetS in both genders. Namely, LAP readings of 26.35 or greater in males and 31.04 or greater in females can help to identify the elderly subjects at high risk of MetS.

## Abbreviations

AUC: Area under the receiver operating characteristic curve
BMI: Body mass index
CVD: Cardiovascular disease
DBP: Diastolic blood pressure
FPG: Fasting plasma glucose
HDL-C: High-density lipoprotein cholesterol
LAP: Lipid accumulation product
MetS: Metabolic syndrome
ROC: Receiver operating characteristic curve
SBP: Systolic blood pressure
TG: Triglyceride
VAI: Visceral adiposity index
WC: Waist circumference
WHtR: Waist-to-height ratio
